# Preschool Environment: Teacher Experiences during the COVID-19 Pandemic in Ghana

**DOI:** 10.3390/ijerph19127286

**Published:** 2022-06-14

**Authors:** Cecilia Obeng, Salome Amissah-Essel, Frederica Jackson, Emmanuel Obeng-Gyasi

**Affiliations:** 1 Department of Applied Health Science, School of Public Heath, Indiana University, Bloomington, IN 47405, USA; fjackso@iu.edu; 2 Department of Health, Physical Education and Recreation, University of Cape Coast, Cape Coast TF0494, Ghana; salome.amissah-essel@ucc.edu.gh; 3 Department of Built Environment, North Carolina A&T State University, Greensboro, NC 27411, USA; eobenggyasi@ncat.edu; 4 Environmental Health and Disease Laboratory, North Carolina A&T State University, Greensboro, NC 27411, USA

**Keywords:** COVID-19, Ghana, preschool, pandemic, health

## Abstract

Background: In Ghana, the COVID-19 pandemic led to the government’s decision to shut down schools for nearly nine months. This study explores the experiences of preschool teachers in Ghana during the COVID-19 pandemic. Methods: The study was carried out using the Qualitative Description approach and aspects of Albert Bandura’s Social Learning Theory. Twenty-five teachers agreed to carry out face-to-face interviews with the researchers. An audio recorder device was used to record the interviews, with each interview lasting between 35–55 min. The analysis was carried out by two researchers who served as coders, and MAXQDA 2022 (VERBI Software GmbH) was used to do the analysis. Results: All twenty-five participants indicated their awareness of COVID-19. Participants said they were so “Scared” when they heard about COVID-19 that it could spell the doom for all humanity. Participants also talked about the extra workload that came upon them as a result of the pandemic and the “financial challenges” that they went through during the pandemic because they had no income since they were not teaching. Study participants indicated that one benefit of the pandemic was the heightened awareness of the need to practice hygienic behavior in their classroom. Conclusion: Participants’ beliefs about the virus being lethal led to mask wearing and the practice of hygienic behavior. Thus, although the COVID-19 pandemic negatively impacted the emotional and financial status of the studied participants, a positive outcome was the participants’ awareness of the need to practice positive health behavior, which will contribute to the overall health and safety of everyone in the preschool environment.

## 1. Introduction

The COVID-19 pandemic resulted in school closures in many countries, including Ghana, and disrupted the educational process, affecting millions of students [1]. Prior to the COVID-19 pandemic, the provision of adequate early childcare services was a challenge, an issue that was further exacerbated by the COVID-19 pandemic [2,3]. It is estimated that about 350 million children lack access to quality early childhood care services globally [4]. Consequently, too many children engage in dangerous and unproductive activities [5]. Due to the pandemic, the world of work has significantly been altered, causing a shift in all sectors of life, including the early childhood care industry [6]. Furthermore, the COVID-19 pandemic raised new challenges in children’s education and their care and well-being [7].

Globally, the effects of the COVID-19 pandemic in 2020 were evident in many countries, including Ghana, with governments imposing stay-at-home orders and restrictions on social gatherings [8]. Parenting roles significantly shifted and evolved, with remote work and childcare services becoming even more crucial [6,7]. The COVID-19 pandemic brought to the fore the critical role of preschool teachers as essential workers were aware of the fact that almost all of the world’s 2.36 billion children, including those in Ghana, were faced with movement restrictions or were in some form of lockdown [7,9].

Access to quality early childhood care services is a global issue that impacts children of lower and middle-income countries such as Ghana differentially [2]. It is estimated that about 80% of the world’s 350 million children who have an unmet need for quality early childhood care services reside in low- and middle-income countries [2]. Existing research attributes this to socio-economic factors [2,10] and, more recently, school and daycare closures in the wake of the COVID-19 pandemic [11].

Similar to most countries, during the surge of the COVID-19 pandemic, Ghana imposed movement restrictions (lockdown) in places such as schools, offices/workplaces and international borders to contain the spread of the virus [12]. From March through to April 2020, the government implemented some restrictions on social gatherings and the enforcement of social distancing guidelines [12]. The most impactful policy implemented during the pandemic was the government’s decision to keep schools closed for roughly nine months of the year [2]. Although parents/guardians were permitted to return to work with adherence to COVID-19 protocols and guidelines (mask-wearing and social distancing), children were confined to the home [2,13]. Despite the grave need for childcare during the pandemic, most early childhood care services in the country remained closed, with many preschool teachers feeling the economic impact of the mandatory closures [14].

This study explores the experiences of preschool teachers in Ghana during the COVID-19 pandemic and the impact of the pandemic on the preschools’ environment or ecology. Our study specifically assessed (a) teachers’ awareness of the COVID-19 pandemic and their sources of information about the pandemic, (b) their feelings during the pandemic and its implications in their lives (c) their perceptions of adjustments brought forth by the pandemic, (d) their personal beliefs about the virus and prevention techniques, (e) their perceptions of their children’s safety in the school environment and (f) the financial implications of the pandemic on their lives.

## 2. Materials and Methods

This research took place after approval from an institutional human subject review board. The questionnaire was developed within the *Qualitative Description* (QD) approach. QD presents descriptions of participants’ narratives [15]. We asked the studied participants to describe their personal experiences in their own words and to suggest ways to improve outcomes or change behaviors [16]. Unlike in other qualitative designs such as phenomenology, ethnography and grounded theory, where the researcher interprets participants’ responses within a context, in QD, the researcher stays close to the data by showing how participants themselves interpret what they say and how relevant their utterances are to them within their speech and environmental domains. Thus, use of the QD design helps us (the researchers) to present rich information grounded in participants’ cultural and environmental contexts. To determine whether the pandemic had any effect on the learning environment (the teacher’s classrooms), we employed aspects of Albert Bandura’s (1977) *Social Learning Theory* of observations and imitation [17]. Albert Bandura explained how children learn in social environments by observing and then imitating the behavior of others. The theory says that children’s learning is affected by factors present in the school environment that influences their behavior.

In this study, the research questions covered the studied participants’ experiences during the COVID-19 pandemic and the arrangements they made in the preschools’ environment to ensure environmental safety.

Twenty-five teachers consented to participate in the study after permission was granted by the Head Teachers of their preschools. A workable schedule was agreed upon with each teacher and face-to-face interviews were conducted at the schools. A Panasonic audio recording device was used to record the interviews. Each interview lasted between 35–55 min.

### Data Analysis

The audio-recorded interviews were transcribed verbatim, and to ensure confidentiality, pseudonyms were assigned to participants. MAXQDA 2022 (VERBI Software GmbH, Berlin, Germany) was used to manage the data during the analysis phase. Specifically, MAXQDA 2022 (VERBI Software GmbH, Berlin, Germany) was used in the organization and analysis of the data, given its well-known capability of helping to achieve the best or most favorable way of data analysis after coding. Working with MAXQDA 2022 helped to create records for each important theme and sub-theme, fill them with the established codes, as well as with participants’ verbatim renditions of their experiences and overall summaries. In particular, the analysis was carried out by the two researchers who served as coders. To familiarize themselves with the content of each transcript, the coders first read and reread the entire transcript meticulously [18]. Then, with an inductive coding approach, the coders identified “significant statements” associated with the research questions and gave codes to those statements [19] in the MAXQDA software. Each statement was first highlighted and dragged into the desired code created in the software. The statements were then grouped under themes. Member checking was carried out to assess if the themes correctly reflected teachers’ experiences during the COVID-19 pandemic. The results are presented below:

## 3. Results and Elucidations

From Table 1, we observe that all the participants 25 (100%) were females. Most of the participants, 16 (64%), were within the age range of 18 and 29 years. Additionally, 12 (48%) of the participants were holders of Diploma Certificates (Associate Degree) in Education. Regarding the number of children in the participants’ classes, half of the participants had class sizes of 21–30.

**COVID-19 Awareness**

All twenty-five participants indicated their awareness of COVID-19. They were aware that it is a deadly disease caused by a virus and that is contagious. Additionally, they were aware of ways to protect themselves from the disease. Excerpts 1 and 2 represent what some of the participants said:

Excerpt 1

*COVID-19 is a deadly pandemic virus that came about early 2019, and then we [in Ghana] got to know it very well in 2020, when we started the first term academic year.*

(p. 13)

Excerpt 2

*I heard that COVID 19 is something that has come to stay with us, a form of a disease and then we have precautions and measures to take to be free from that pandemic. Some of them are after going out, when you are back; wash your hands under running water, space out when you are in a crowded area.*

(p. 23)

From Excerpts 1 and 2, we are led into the participants’ perception of the deadly nature of the virus, its inception, when they became aware of it and the need to take precautionary measures such as handwashing and social distancing to mitigate or deal with its spread.

All the participants indicated that they received information about the COVID-19 pandemic from experts mostly through the mass media, especially radio and television. The quotes in Excerpts 3 and 4 illustrate some of the participants’ accounts.

Excerpt 3

*Oh, for expert, even in our school, you know, our district, there are nurses that come here to organize weighing, when they come, they talk to us, but before then, on TV, on radio, everywhere, you hear the health experts talking, the Minster of Health, the Director of Health, everybody is talking. When we even go to church, they come there to talk to us.*

(p. 3)

Excerpt 4

*Okay so personally, we have friends who are health workers, so always they give us information. Information was on the TV, on radio, on the internet, so basically, information was everywhere about COVID-19.*

(p. 4)

Close observation of the above excerpts points to the various sources of information regarding the pandemic. The sheer number of experts and sources that provided information about the pandemic—ranging from school nurses, district directorate of education, TV, radio, the Minister of Health, Director of Health, health workers and internet sources—point to a broad and a serious and/or intensive health education program about the pandemic.

**Feelings about COVID-19 in Ghana**

Two themes emerged out of the narratives of the participants concerning how they felt when they first heard that COVID-19 cases had been recorded in the country. The themes are: *Anxiety* “Scared” and *Doom* “End of the world”.

**Anxiety** *(Scared)*

Participants expressed various degrees of anxiety (fear) falling on everybody when the disease was first recorded in Ghana. People were very scared. Some had this to say:

Excerpt 5

*I was really scared. Initially, we heard it was in countries outside Ghana but when it came to Ghana, I was scared…. in fact fear fell on everyone. As we were seeing in documentaries, the way people were dying it was scary.*

(p. 25)

Excerpt 6

*I was scared. Sacred of getting some, may be someone close to me may get some and I will also get it. So I was scared when it came to Ghana.*

(p. 23)

From Excerpts 5 and 6, we observe anxiety on the part of the studied participants. Even though only two excerpts are cited here for exemplification, this theme ran across the entire data. Participants’ anxiety was captured in the word *scared*. The use of the quantifier *everyone* points to the extent of the fear among the participants and the overall community in which they lived.

**Doom** *(End of the world)*

Another perspective that came up regarding how participants felt about the COVID-19 pandemic was doom; the religious idea that it was God who was punishing humans for their sins with the possibility of the world coming to an end. Two participants shared this in Excerpts 7 and 8 below:

Excerpt 7

*I have seen how over the years we celebrate Afahye (festival) and so I just said to myself that……. because of what we were doing that is why God is punishing us, bringing COVID to destroy some things. So, I know that it’s a disease, but God too is using that to punish us, because human beings, we believe that there is God.*

(p. 7)

Excerpt 8

*From the way things were going, I thought the world had come to an end. Because no matter your age, whether rich, poor or whatever, it [COVID-19] just catches you and takes you off.*

(p. 20).

From Excerpt 7 we observe a participant attributing the pandemic to the celebration of a traditional festival (which in her opinion was fetish or an act of godlessness) hence making God angry and consequently making him punish people for their disloyalty and disobedience. In Excerpt 8, the participant saw the virus as not being a respecter of persons; that is, attacking everyone irrespective of their socio-economic standing or age hence an indication of the end times. In both excerpts, we observe an entwinning of religion and health from the participants’ perspectives.

**New arrangements in their preschool classrooms as a result of COVID-19**

All twenty-five participants indicated that their preschool classrooms have had to undergo new arrangements or restructuring spatially as a result of the pandemic, particularly as a result of following the COVID-19 protocols prescribed by the Government of Ghana. Two excerpts (9 and 10) are cited below for exemplification and elucidation.

Excerpt 9

*So now, we have spaced out the children in the classroom, the sitting arrangement has changed. Formerly, they sit round a table, but we have tried to separate them, so that we can keep to the social distancing protocol rule. We also ensure that before they enter the classroom, they wash their hands; they are in their nose masks and all that.*

(p. 4)

Excerpt 10

*When a parent brings a child to school, we check the temperature of both of them after that, their hands are sanitized. The teachers also sanitize their hands before taking the children to the class. We also wash the children’s hands before they eat their food and after eating. We also use disinfectant to clean the class before and after closing, so that the disease can not affect them.*

(p. 5)

Both Excerpts 9 and 10 deal with action taken by the teachers to make the learning environment safe with the aim of controlling the spread of the virus. Actions such as social distancing, handwashing, hand sanitizing, temperature checking and disinfecting are all standard practices known to stop or mitigate the spread of the COVID-19 virus.

Participants also explained why it was important for them to do the new arrangements in their classrooms. The following quotes (Excerpts 11 and 12) illustrate the participants’ stories.

Excerpt 11

*We did social distancing to avoid the spread of the COVID because we didn’t know anyone who is having it. It is not written on anyone’s forehead to know that this person is having COVID whereby you will isolate the person from the people. So, because of that we give the spacing to avoid the contact between them.*

(p. 9)

Excerpt 12

*We did all this to make sure that the children are safe in the school. Because during the early stages, some of the parents were afraid to bring their children because they thought in the schools the teachers may be will not be able to protect them so because of that we had to make sure that the parents see us doing all the protocols.*

(p. 22)

From the above excerpts, it was clear that the studied participants undertook the above-mentioned actions as precautionary measures to ensure the children’s safety and to create conducive and healthy learning environments.

Next, we present the way and manner in which the studied participants’ beliefs about COVID-19 shaped their health-related activities in the classroom ecologies.

**How beliefs about COVID-19 influenced teachers’ work**

The narratives of the participants concerning how their beliefs about COVID-19 influenced their work in the preschool classrooms are grouped under two thematic areas: “personal belief influences” and “parental belief influences”.

***Personal belief influences***

Participants indicated that their personal beliefs about COVID-19 led them to take extra precaution in their workplace environment. The following two excerpts from their narratives give credence to the above assertion.

Excerpt 13

*Personally, as I said earlier, the virus was deadly so I was afraid that I will die and I did not want the kids too to be dying or suffering from this virus, so I always wear my nose mask, practice social distancing, ensure that they all wash their hands, wear their nose mask and we will all be safe.*

(p. 2)

Excerpt 14

*The belief I had about the COVID was that the disease was not there. They will say that there are recorded cases in my area but no one was seeing the recorded cases. However, my belief changed when the owner of the school kept on telling us that COVID 19 was real. Before COVID, we were not wearing mask to school but now we do. Also we use to use one uniform to school but now because of COVID, a room has been allocated to staff to change our dress and shoes we wore from home and put on uniforms kept in the school.*

(p. 24)

In Excerpt 13, we learn that teachers’ personal beliefs about the virus and fear of the virus as well as their professional obligation to keep the children safe (by helping to protect them from the deadly virus) led them (the teachers) to wear their masks, socially distance, practice handwashing and to ensure that the children to do the same.

In Excerpt 14, the participant mentions her doubt about the authenticity or reality of the virus and a change in belief when the Principal of the school continually emphasized the virus’ reality. She then explains a change in the teaching faculty members’ precautionary action involving dressing protocol prescribed by the school. What is significant here is the school authority’s obligation in educating faulty and its power over the faculty regarding decisions about safety.

***Parental belief influences***

Participants also indicated in their narratives how some parental beliefs about COVID-19 led them to be extra cautious in their workplace. The following quotes (Excerpts 15 and 16) exemplify the above assertion.

Excerpt 15

*Yes; most of them [parents] were adamant to bring their kids to school, because they believe, these ones are too young, so how can they say they will not put their hands in their nose, they can take someone’s nose mask and wear if theirs get missing, so most of them were not bringing their kids to school. Even when the government asked us to go back to school, most of them were staying in the house, so in fact, it really affected our teaching.*

(p. 19)

Excerpt 16

*Yes. I quite remember a Lecturer who has a child here came and a Teacher, I don’t know whether the teacher accidentally refused to wash the hands and the lecturer confronted her that even the children are washing their and you refused to wash yours and the teacher apologized and said “I was late so I was in a hurry to go to my class so pardon me for that. So the teacher had to come back and wash the hands before going to the class. So we are all careful to follow the protocols strictly”.*

(p. 22)

From Excerpts 15 and 16, we observe parents’ beliefs about children’s ‘normal’ behaviors or practices regarding nose touching, wearing others’ masks, among others, posing as a risk to the children’s safety. These beliefs led some parents to decide to keep their children at home thereby creating unintended school attendance problems. A parent also observed a faculty member not following the standard hygiene protocol and expressed dissatisfaction and urged the faculty member to wash her hands. Thus, we see from the narratives the nature of parents’ beliefs about the pandemic and the extent to which such beliefs impacted teachers’ work and the overall classroom ecology.

**Work experiences during the COVID-19 Pandemic**

Four themes were identified in the narratives of the participants concerning their work experiences during the pandemic. The themes are: “Financial challenges”, “COVID-19 protocol challenges”, “Workload challenges” and “Unintended benefits” or “The good side” of the pandemic.

***Financial challenges***

Participants talked about various financial challenges they went through during the pandemic. Excerpts 17, 18 and 19 illustrate participants’ accounts.

Excerpt 17

*What I can also say is that, the disease disturbed us a lot, because during the lockdown, I was taking half payment which did not help me at all.*

(p. 5)

Excerpt 18

*COVID has come to disturb us. During the lockdown because we were not working, our pay, our finance……everything went down in the sense that this is a private school and we are paid from the fees of the children so if children are not coming to school it means no fees paid which also meant that we the teachers could not be paid……However, by the grace of God now things are a little resolved.*

(p. 25)

Excerpt 19

*Look at the school we find ourselves, private institution, some [parents] were even scared to pay the school fees, because they had the belief that the government will definitely close the schools……and nobody will refund the fees……… I had to move from one house to the other to go and do home teaching that I will also get something for my kids and my household, so living was very very tough.*

(p. 20)

From Excerpts 17 to 19, we learn about the financial hardship the teachers went through and the difficulty in meeting their financial obligations due to either non-payment of salaries or salary cut. We also highlight the research participants’ coping strategies, some of which involved giving private in-home tuition (teaching), as fear of the unknown led to anxiety regarding the steps the government could take regarding school closure and its adverse financial implications for them. Next, we look at challenges related to the COVID-19 protocols.

***COVID-19 protocol challenges***

In the participants’ narratives, it emerged that they were faced with challenges following the COVID-19 protocols. They shared these below.

Excerpt 20

*Work is somehow difficult, because before the COVID and now, our interaction with the children has changed. There are certain things you can’t do, sometimes even when you want to hold a child’s hands or book, you are afraid, because you don’t know where the child is coming from and it is very very difficult coping with. Using sanitizer, sometimes I don’t even like the scent of the sanitizer, but because of COVID, I have to use it.*

(p. 13)

Excerpt 21

*I will say it is very very tedious, because handling kids who should be in mask for some number of hours, students who like playing now being restricted not to play with their friends again and handling them is a tedious work, but as time goes on, it is becoming somehow easy to manage. But at the beginning it was very tiresome and then very stressful.*

(p. 15)

Excerpts 20 and 21 above exemplify and elucidate the difficulties that the teachers went through in helping the children become aware of the COVID-19 protocols regarding hand sanitizers, handling children’s books or even holding their hands. Fear of not knowing the children’s home approach to virus prevention meant that the teachers either had to take risks and hold the children, their books among other things. Mask wearing had a tiring and tedious effect on both the children and the teachers making classroom management difficult. In effect, the pandemic created a professional quagmire for the teachers who had no option other than find ways to assist the children.

In the next section, we take a close look at the workload challenges the teachers endured during the pandemic.

***Workload challenges***

In the narratives of the participants, they talked about the extra workload that they experienced as a result of the pandemic. Excerpts 22 and 23 illustrate the participants’ challenges.

Excerpt 22

*Work experience, as I initially said, schools closed down and so we didn’t go to school for a while, but when we came back in January, it has impeded the work in a while. You know these people are children between 4, 5, 6 years and sometimes you are saying recitals, you want them to also say it, and because they are in mask, you don’t know whether the child is saying it or not, and even you the teacher, you have to strain your voice and shout, because you are in mask, you cannot take off your mask when you are teaching.*

(p. 4)

Excerpt 23

*COVID has handed to us another workload, because aside classroom teaching, we are now obliged to make sure pupils are well taken care of, they don’t go out and stay for long, they wash their hands frequently, they put on their masks and sanitize their hands frequently …. So it has made work a little more difficult, because these are things we are not used to, we are trying to adjust ourselves, so my experiences so far, it’s not easy, but we are trying.*

(p. 14)

Excerpts 22 and 23 exemplify and elucidate the difficulties the teaching faculty encountered in the performance of their professional duties as educators. Specifically, the teachers encountered challenges related to auditory and/or perception, as well as sound articulatory and acoustic challenges due to the wearing of masks that impeded speaking and perception. Participants also complained about extra workload relating to caregiving, handwashing and hand sanitizing. This extra workload overwhelmed the faculty. The Ghanaian English expression, *it’s not easy, but we are trying*, points to the faculty being overwhelmed but having to endure due to either their love for their profession or the lack of a better job alternative.

In the next sub-section, we focus attention on handwashing given its frequency of occurrence in the narratives and its importance to the classroom ecology.

***Hand Washing***

Participants narrated how despite the fact that the COVID-19 pandemic posed a great challenge to them, it also led them to acquire a positive behavior in their classrooms, related to children washing their hands all the time. The studied participants indicated that this positive behavior contributed to the health and safety of everyone in the preschool environment. The following four excerpts illustrate the participants’ accounts.

Excerpt 24

*I make sure that I wash my hands before I take any child,*

*The children also since they see us washing our hands all the time, they also wash their hands all the time. This kind of hand washing is helping all of us.*

(p. 1)

Excerpt 25

*In fact the children are washing their hands often since they see us washing our hand.*

(p. 3)

Teachers also reported about the increased use of hand sanitizers in their classrooms during the COVID-19 pandemic. They said some of the children asked their parents to buy them hand sanitizers.

Teachers indicated:

Excerpt 26

*Some of the children had their personal hand sanitizer which they used all the time because they saw the teachers using the hand sanitizer.*

(p. 4)

Excerpt 27

*The children ask me for hand sanitizer or washed their hands because they see all the teachers washing their hands or using hand sanitizer.*

(p. 5)

The above excerpts (24–27) highlight that the studied participants and their school children became attached to personal hygiene (in the areas of handwashing and hand-sanitizing) due to the pandemic. This unintended benefit, a teacher noted, *is helping all of us*. Thus, the lesson on hygiene, learned as a result of following the protocols set by the government and the schools, benefited both the teachers and children.

Next, we carry out a general discussion of our research findings by recapitulating and extending some of the facts explicated in this section and then relating them to the literature. This will be followed by limitations of the study and a brief conclusion in Section 5.

## 4. General Discussion

The COVID-19 pandemic in Ghana has had a significant impact on the school environment. Our study indicated that participants were aware of COVID-19 and obtained most of their information from experts through the mass media. Studies have suggested that the sources of information about the pandemic determine one’s perceptions and views. Thus, messaging is critical in limiting misinformation [20,21]. Our study thus bolsters the findings of the above studies given the fact that the studied participants noted that their course of action in their classrooms was based on information they obtained from experts mainly via the mass media.

Participants were generally anxious, fearful and scared when watching and reading the news surrounding the pandemic. This fear is partly because the ongoing pandemic is a novel one, and experts are still learning about it and how it is transmitted and treated in populations. In addition, research has shown that uncertainty about COVID-19 can bring about fear [22]. Once again, our findings are in line with and support those of other experts thereby giving credence to psychological and/or emotional challenges associated with the unknown or about health issues, about which knowledge is inadequate.

Some of the studied participants felt the pandemic indicated an end to the world. This view, as noted earlier, partly stems from the strong Christian belief in Ghana which speaks of the end times where unexplained diseases will ravage the world, with the righteous eventually earning a ticket to Heaven. This view is not unique to our studied participants; similar findings that speak of COVID-19 and its underling apocalyptic connection are also documented in the literature [23].

In this study, schools had decided to adjust to the new circumstances that the COVID-19 pandemic had created. These adjustments were to limit the spread of COVID-19. Some spoke about parents’ fears of children coming home after being in the school environment. This concern matches the work of others who have demonstrated that fear exists that children can serve as non-symptomatic carriers of the disease and subsequently serve as a source of exposure [24]. However, a systematic review indicated that household transmission studies revealed that children were seldom the index case and children with COVID-19 rarely triggered outbreaks [25].

Participants’ personal beliefs about the virus being deadly and mask wearing were seen in this study. It must be noted that some felt that news of the virus was overblown as no cases were seen within their community, and this influenced their perceptions, while others were very fearful and acknowledged the dangers the virus posed. The dichotomy of believing the virus is either overblown or extremely dangerous has been seen in many other environments but may offer different insights in the West African context. The pandemic has not been particularly bad or caused disastrous consequences in this region [26], and the population structure tends to be younger. In addition, the presence of less testing means that milder cases in a younger population may not necessarily indicate to those in less-affected parts of the region that the virus is a present threat.

Parental beliefs that children lacked the agency to protect themselves against the pandemic led some not to want them to be in the school environment. In addition, children’s behavioral patterns, such as hand-to-face behaviors, make them more prone to exposure [27]. In the context of the severe acute respiratory syndrome coronavirus 2 (SARS-CoV-2), because the consequences are not as profound in children, the parental concern may be viewed in the context of spreading it to adults but also may be considered in the context of the lack of extensive information about its short-, medium- and long-term consequences in children [28].

Financial challenges characterized the work experiences during the COVID-19 pandemic. As a result, some had their salaries significantly decreased. Financial hardship brought forth by the pandemic offers unique challenges in a low-middle-income country (LMIC) where the safety net for families is not as extensive as in some more developed countries. In the context of Ghana, though safety nets exist, access to them is characterized by vast bureaucracy, with many also unaware of their existence [29].

The COVID-19 protocols presented significant challenges to the teachers. Firstly, masking diminishes the verbal human interaction between individuals and may hamper communication. In addition, the fear caused other non-verbal common behaviors such as declining handholding due to the fear of spreading the virus. These non-verbal communication tools are critical for children’s development and social skills [30]. In countries such as Ghana, where such communication is standard, diminishing it may hamper health in the immediate and longer term [31].

Workload challenges during the pandemic were also a challenge. For example, teachers were expected to monitor and teach hygienic behavior to children. Although the extra workload of teachers in the classroom for younger children has been demonstrated in the literature [32], this workload increase in the context of a global pandemic of a novel virus means that this burden was dynamic and could increase stress and anxiety [33].

This study also indicated a benefit of the pandemic, which is heightening awareness of the need to practice hygienic behavior. The literature has revealed that people are now more conscious of their hygiene [34]. Increased knowledge of viruses, how they are transmitted and basic preventative methods to put in place plays a significant role in diminishing the prevalence and incidence of other infectious diseases [35]. In addition, these behaviors may reduce exposure to toxicants such as heavy metals from dust and air pollutants [36].

### Limitations

Although the researchers used similar research questions from their previous COVID-19 research, the questionnaire was not pilot tested to see whether it was appropriate for our studied participants. Additionally, the data analysis focused solely on the recorded interview; we did not go back to ask further questions. Another limitation is that the study participants were women only. However, the *Qualitative Description* approach enables us to obtain rich data from the study participants and set the foundations for more extensive studies to be completed.

## 5. Conclusions

This study has enabled us to explore the experiences of preschool teachers in Ghana. Delving into participants’ experiences demonstrated that they were fearful and scared of the pandemic. Their beliefs about the virus being lethal led to mask wearing and the practice of hygienic behavior, which also contributed to the children imitating the handwashing behavior of the teachers. The behavior imitation of the children is in agreement with Bandura’s theory which indicates that children imitate the behavior of people around them and behave in a similar manner. In conclusion, based on the participants’ narratives, it is true to say that although the COVID-19 pandemic had a tremendous impact on the participants’ finances, workload and interaction with children, it also encouraged them to develop positive behaviors such as handwashing which has implications for health education. Teachers should use the handwashing skills acquired through this pandemic period to teach children about the importance of handwashing in children’s daily lives. This will help children’s health and will contribute to the health and safety of everyone in the preschool environment. Future research will target men’s experiences during the COVID-19 pandemic in the preschool environment.

## Figures and Tables

**Table 1:** Demographic characteristics of the participants.

Teacher Characteristics	Frequency	Percentage
Gender		
Female	25	100
Age		
18–29	16	64
30–39	6	24
40–49	1	4
50–59	2	8
Educational Qualification		
High School	5	20
College of Education Diploma	12	48
First Degree	7	32
Class Size		
10–20	7	28
21–30	13	52
31–40	3	12
41–50	2	8

## Data Availability

The data will be made available upon reasonable request.
